# Finding Treatment Effects in Alzheimer Trials in the Face of Disease Progression Heterogeneity

**DOI:** 10.1212/WNL.0000000000012022

**Published:** 2021-06-01

**Authors:** Roos J. Jutten, Sietske A.M. Sikkes, Wiesje M. Van der Flier, Philip Scheltens, Pieter Jelle Visser, Betty M. Tijms, for the Alzheimer's Disease Neuroimaging Initiative

**Affiliations:** From the Department of Neurology, Alzheimer Center Amsterdam, Amsterdam Neuroscience, Amsterdam UMC (R.J.J., S.A.M.S., W.M.V.d.F., P.S., P.J.V., B.M.T.), and Clinical Developmental Psychology & Clinical Neuropsychology (S.A.M.S.), VU University; Alzheimer Center Limburg, School for Mental Health and Neuroscience (P.J.V.), Maastricht University, the Netherlands; and Department of Neurobiology, Care Sciences and Society, Division of Neurogeriatrics (P.J.V.), Karolinska Institutet, Stockholm, Sweden.

## Abstract

**Objective:**

To investigate the influence of heterogeneity in disease progression for detecting treatment effects in Alzheimer disease (AD) trials, using a simulation study.

**Methods:**

Individuals with an abnormal amyloid PET scan, diagnosis of mild cognitive impairment or dementia, baseline Mini-Mental State Examination (MMSE) score ≥24, global Clinical Dementia Rating (CDR) score of 0.5, and ≥1 follow-up cognitive assessment were selected from the Alzheimer's Disease Neuroimaging Initiative database (n = 302, age 73 ± 6.7; 44% female; 16.1 ± 2.7 years of education; 69% *APOE* ε4 carrier). We simulated a clinical trial by randomly assigning individuals to a “placebo” and “treatment” group and subsequently computed group differences on the CDR–sum of boxes (CDR-SB), Alzheimer’s Disease Assessment Scale–cognitive subscale–13 and MMSE after 18 months follow-up. We repeated this simulation 10,000 times to determine the 95% range of effect sizes. We further studied the influence of known AD risk factors (age, sex, education, *APOE* ε4 status, CSF total tau levels) on the variability in effect sizes.

**Results:**

Individual trajectories on all cognitive outcomes were highly variable, and the 95% ranges of possible effect sizes at 18 months were broad (e.g., ranging from 0.35 improvement to 0.35 decline on the CDR-SB). Results of recent anti-amyloid trials mostly fell within these 95% ranges of effect sizes. *APOE* ε4 carriers and individuals with abnormal baseline tau levels showed faster decline at group level, but also greater within-group variability, as illustrated by broader 95% effect size ranges (e.g., ±0.70 points for the CDR-SB).

**Conclusions:**

Individuals with early AD show heterogeneity in disease progression, which increases when stratifying on risk factors associated with progression. We provide guidance for a priori effect sizes on cognitive outcomes for detecting true change, which is crucial for future AD trials.

Primary endpoints in Alzheimer disease (AD) trials are usually slowing or halting cognitive decline, which is typically evaluated using neuropsychological tests or scales that capture global cognitive functioning.^1,2^ A crucial assumption here is that the treatment and placebo group should show similar rates of cognitive decline in the absence of a treatment effect.^3^ However, several longitudinal studies have suggested that individuals with AD show considerable heterogeneity in their rates of cognitive decline, even when matched on disease severity at the start of the study.^4–7^ Randomization procedures, however, do not necessarily result in equal rates of cognitive decline between treatment and placebo groups (assuming rates exceed background noise).^8^ A consequence is that placebo vs treatment group differences on cognitive outcome measures may thus depend on variation in sampling of slow vs fast decliners (figure 1). It remains unclear how such variation in random sampling would influence trial outcomes, and may explain recent trial failures^9–14^ or tentative successes.^15,16^

**Figure 1:**
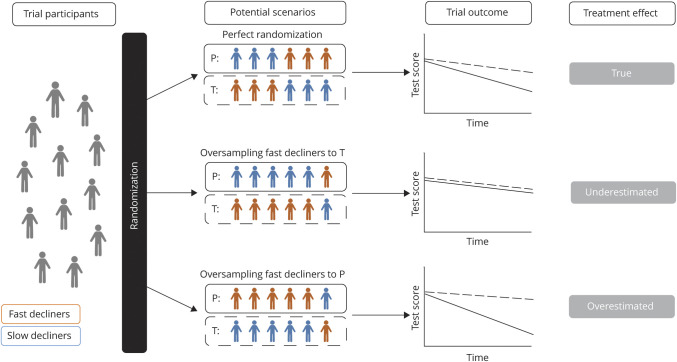
Hypothetical Examples of Potential Effect Sampling Effects on Trial Outcomes These examples show how random oversampling or undersampling of slow vs fast decliners could affect trial outcomes when rate of cognitive decline is not accounted for during randomization. We depict 2 extreme possible situations of oversampling in order to illustrate the influence of an imbalance between slow and fast decliners compared to a perfect randomization. P = placebo group (reflected by solid line); T = treatment group (reflected by dotted line).

We studied the effect of heterogeneity in progression on cognitive outcome measures in individuals from the Alzheimer's Disease Neuroimaging Initiative (ADNI) who met inclusion criteria that are currently used in prodromal and mild AD trials.^17^ We investigated the variability in effect sizes that can be observed between a “placebo” and “treatment” group after 18 months when heterogeneity in progression is unaccounted for, and compared this to effect sizes that have been reported for recent anti-amyloid trials.^9,10,12,14,15^ We further studied the influence of known factors affecting AD progression (i.e., age, sex,^18^ educational level,^19^ *APOE* ε4 status,^20^ CSF tau levels^4^) on the variability in effect sizes.

## Methods

### Study Participants

Data were obtained from the ADNI research database (adni.loni.usc.edu/wp-content/uploads/2008/07/adni2-procedures-manual.pdf). ADNI is a multicenter longitudinal cohort study with the primary goal of testing whether serial neuroimaging and other biological, clinical, and neuropsychological markers can be combined to measure clinical progression on the AD spectrum. Data for the current study were selected from the ADNI-1, ADNI-GO, ADNI-2, and ADNI-3 phases. We selected those individuals who had at least 1 year clinical follow-up available and met the inclusion criteria that were used in the recent EMERGE and ENGAGE trials^17^: (1) a clinical diagnosis of mild cognitive impairment (MCI) or dementia; (2) elevated amyloid as measured by PET imaging (specific procedures and cutoff described below); (3) an MMSE score ≥24 at baseline; and (5) a global Clinical Dementia Rating (CDR) scale of 0.50 at baseline.

### Standard Protocol Approvals, Registrations, and Patient Consents

Data used in the current study were collected between February 2007 and November 2019. The study protocol was approved by an ethical review board and all participants provided written informed consent.

### Data Availability

All data used in this article are publicly available and were downloaded from the ADNI website (adni.loni.usc.edu). We will provide a list of ADNI participant identifications for replication purposes upon request.

### Measures

#### Cognitive Outcome Measures

We searched ClinicalTrials.gov in February 2020 on AD clinical trials and found that out of the 208 registered phase 3 trials, 149 (72%) reported the use of cognitive outcome measures in an anti-amyloid treatment. The top 3 most used were the Alzheimer's Disease Assessment Scale–cognitive subscale (ADAS-Cog; n = 74 studies),^21^ the Mini-Mental State Examination (MMSE, n = 58 studies),^22^ and the CDR scale–sum of boxes (CDR-SB; n = 46 studies).^23^ Therefore, we took these outcomes (i.e., ADAS-Cog, MMSE, and CDR-SB) to study cognitive change.

The CDR was originally developed for the staging of dementia severity. The participant's cognitive and functional performance is rated in 6 areas: memory, orientation, judgment and problem-solving, community affairs, home and hobbies, and personal care. Each area is rated as 0 (“healthy”), 0.5 (“questionable dementia”), 1 (“mild dementia”), 2 (“moderate dementia”), or 3 (“severe dementia”). Adding the rating of all boxes results in a total sum of boxes (CDR-SB) score ranging from 0 to 18, with higher scores reflecting worse impairment.^23,24^ The 13-item version of the ADAS-Cog (ADAS-Cog-13) yields a measure of cognitive performance by combining ratings of 13 subtests that mainly focus on episodic memory, praxis, and language (e.g., word lists recognition and recall, constructional praxis, object and finger naming) domains. Total scores range from 0 to 85, with higher scores indicating worse impairment.^21^ The MMSE is a global cognitive screening test, with total score ranging from 0 to 30, and lower scores reflect worse impairment.^22^

#### Biological Markers

Amyloid positivity was determined by Pittsburgh compound B (PiB) or Florbetapir AV-45 PET scanning (summary data were obtained from the ADNI Laboratory of Neuroimaging database: loni.ucla.edu/ADNI/). Amyloid abnormality was based on standardized uptake*value*ratio of mean uptake in 4 cortical regions (frontal, cingulate, parietal, and temporal cortices) normalized to the whole cerebellum uptake, using validated tracer-specific cutoff values (>1.10 for Florbetapir and >1.47 for PiB-PET).^25^ Tau was measured in CSF with the xMAP Luminex platform (Luminex Corp.) and dichotomized into normal and abnormal based on a cutoff value of 93 pg/mL.^26^ *APOE* genotype was dichotomized into individuals carrying at least 1 *APOE* ε4 allele (i.e., carriers) or none (i.e., noncarriers).

### Statistical Analyses

**Figure 2:**
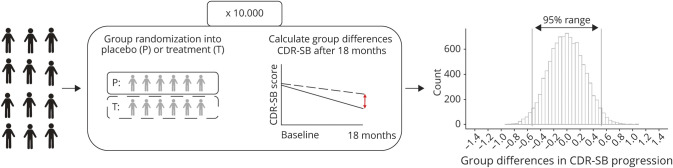
Schematic Overview of Simulation Procedure CDR-SB = Clinical Dementia Rating scale–sum of boxes; P = placebo group, reflected by solid line; T = treatment group, reflected by dotted line.

Statistical analyses were performed using R version 3.5.3 (R Core Team, 2018). We modeled effect of oversampling or undersampling of fast/slow decliners on the variability in effect sizes observed on the ADAS-Cog-13, MMSE, and CDR-SB, using the following procedures for each cognitive test. First, we modeled cognitive decline by running linear mixed models (LMMs) with random intercepts and slopes for each subject using the cognitive test scores as dependent variable and time (measured on a continuous level and including all available follow-up time points) as independent variable. For individuals with longitudinal assessments, but without an observed 18-month assessment, we used these LMMs to predict their test score at 18 months follow-up. We then computed the baseline to 18 months change score for each individual as input for our simulation analyses. Next, we simulated a clinical trial by randomly dividing the overall group into a placebo and “treatment” group and calculating “treatment effects” by computing group differences in change from baseline to 18 months follow-up. We repeated this simulation 10,000 times to determine the 95% range of observed effect sizes (figure 2), demonstrating the possible range of scores that can be expected when there is no treatment effect and heterogeneity in decline is unaccounted for at baseline. To investigate whether the range of group differences could be explained by differences in demographics, we also simulated group differences in age, sex, level of education, *APOE* ε4 status, and baseline tau levels, and computed Pearson correlations between changes in each of these variables to the group difference in change on cognitive outcome measures.

Subsequently, we compared the 95% range of effect sizes to placebo vs (high-dose) treatment group differences in progression on the CDR-SB, ADAS-Cog, and MMSE that have been reported for ENGAGE^15^ and EMERGE,^15^ as well as the DAYBREAK-ALZ,^12^ IDENTITY-2,^9^ EXPEDITION-3,^10^ and BAPINEUZUMAP^14^ trials, which had similar inclusion criteria and follow-up duration as the ENGAGE and EMERGE trials.

We further performed 3 sensitivity analyses. First, we repeated this simulation procedure including only those individuals with observed 18-months data, and next including only those individuals with an CDR-SB score of 0.50 at baseline. Third, we repeated the simulation with datasets including n = 1,000, n = 2,000, n = 5,000, and n = 10,000 cases who all had simulated 18-months data on the CDR-SB, ADAS-Cog, and MMSE based on the aforementioned LMMs, to investigate the influence of sample size on the variability in effect sizes.

Finally, we studied whether effects of heterogeneity on outcomes would decrease if groups were stratified on known risk factors for AD, i.e., age (cutoff of 65 years), sex (male vs female), education (high vs low, based on a cutoff 15 years), CSF tau levels (normal vs abnormal), and *APOE* ε4 status (carrier vs noncarrier).

## Results

A total of 302 individuals were included (mean age 73 ± 6.7 years; n = 133 [44%] female; mean level of education 16.1 ± 2.7 years; n = 207 [69%] *APOE* ε4 carrier), of whom 274 had MCI and 28 mild dementia at first visit. On average, individuals were followed for 3.8 ± 2.3 years (maximum follow-up time ranging from 1 to 9 years) and had 5 ± 2.1 repeated cognitive assessments (number of repeated assessments ranging from 1 to 11).

### Heterogeneity in Disease Progression

**Figure 3:**
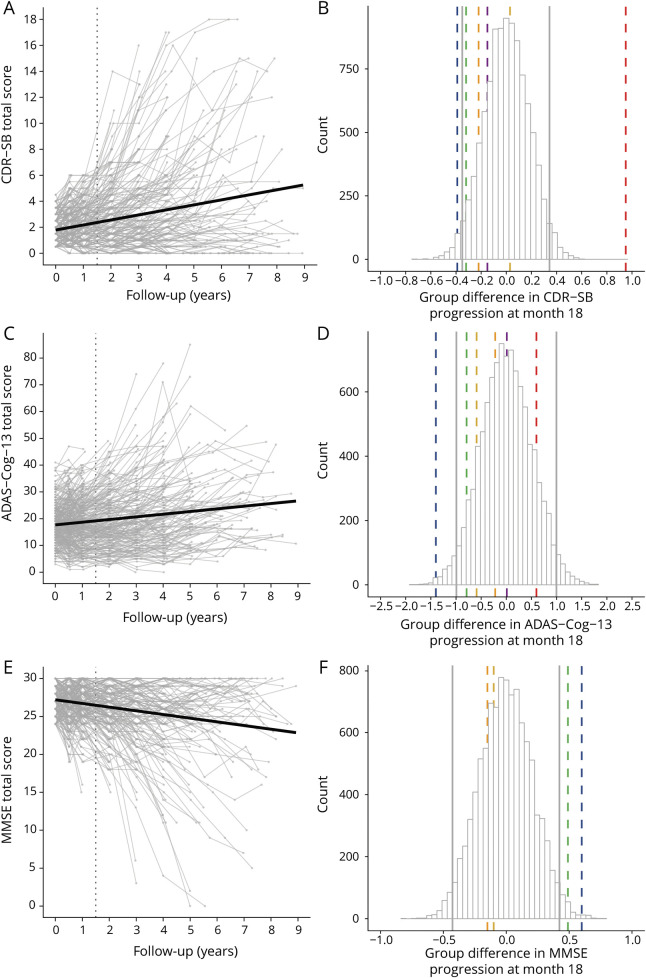
Individual Trajectories and Simulated Group Differences for All Outcome Measures in the Total Sample (n = 302) Left column: Individual trajectories on the Clinical Dementia Rating scale–sum of boxes (CDR-SB) (A), the Alzheimer's Disease Assessment Scale–cognitive subscale (ADAS-Cog) (C), and the Mini-Mental State Examination (MMSE) (E). Dotted vertical line presents scores at the 18 months time point. Right column: Simulated group differences in change from baseline to month 18 based on the total sample (n = 302) on the CDR-SB (B), ADAS-Cog–13-item version (ADAS-Cog-13) (D), and MMSE (F), including 95% range of effect sizes as indicated by vertical gray lines and effect sizes reported for recent clinical trials as indicated by vertical colored lines (blue = EMERGE^15^; yellow = ENGAGE^15^; green = EXPEDITION-3^10^; orange = DAYBREAK-ALZ^12^; red = IDENTITY-2^9^; magenta = BAPINEUZUMAP^14^)*.*

The spaghetti plots in figure 3 show individual trajectories of cognitive decline over time and illustrate that individuals vary greatly, with person-specific slope estimates on the CDR-SB ranging from 0.31 points improvement to 3.73 points worsening per year (mean annual change *+*0.76 points, SE 0.06, 95% CI 0.65–0.88, *p* < 0.001) (figure 3A). Similarly, person-specific slopes on the ADAS-Cog ranged from 1.01 points improvement to 12.55 points worsening per year (mean annual change +2.44 points, SE 0.14, 95% CI 2.08–2.82, *p* < 0.001) (figure 3B), and individual slopes on the MMSE ranged from 0.60 points improvement to 5.80 worsening per year (mean annual change −0.96 points, SE 0.09, 95% CI −1.13 to −0.0.79, *p* < 0.001) (figure 3C).

The 212 individuals (201 MCI/11 dementia, age 73 ± 6.8; 44% female; 16.2 ± 2.8 years of education; 71% *APOE* ε4 carrier) who had observed 18-month data available did not differ in baseline characteristics compared to those without observed 18-month data, and showed similar estimates for person-specific slopes (figure e1, A, C, and E; doi.org/10.5061/dryad.qjq2bvqf2).

### Trial Simulation Results

Figure 3, B, D, and F, shows for each cognitive outcome the range of group differences in change from baseline to 18 months, obtained across 10,000 repetitions of randomly assigned “placebo” and “treatment” groups. Vertical gray lines indicate the lower and upper levels within which 95% of group differences were observed. For the CDR-SB, 95% of group differences fell within a range of −0.35 to +0.35 (figure 3B). For the ADAS-Cog, 95% of group differences ranged from −1.00 to −1.00 (figure 3D), and for the MMSE, this 95% range was −0.42 to +0.42 (figure 3F). Group differences in progression across simulation runs were not associated with group differences in age, sex, level of education, *APOE* ε4, or baseline tau levels (all correlations ranging between *r =* 0.00 and *r =* 0.02).

To place recent trial findings in perspective of our findings, we added trial results on each outcome measure as reported for recent anti-amyloid trials in prodromal and mild AD. Each colored dashed line in the histograms indicates the actual observed differences in cognitive decline between treatment and placebo groups for a specific trial. It can be seen that almost all observed group differences showed improvement of treatment vs placebo as captured by negative group differences on the CDR-SB and ADAS-Cog and positive group differences on the MMSE, but most differences fell within the 95% ranges of our simulation for all outcome measures. Only the blue line, i.e., the EMERGE trial, showed worsening of the placebo compared to the treatment group that was outside the lowest 95% percentile of group differences on all outcome measures. On the other hand, the red vertical line, i.e., the IDENTITY-2 trial, showed a worsening of the treatment group that was outside the highest 99% percentile of group differences for CDR-SB, suggesting that this was unlikely a chance finding (although this effect was not observed for the ADAS-Cog).

#### Sensitivity Analyses

Repeating the analyses including only individuals with observed 18-month data (n = 212) slightly broadened ranges of effect sizes, as shown in figure e1, B, D, and F (doi.org/10.5061/dryad.qjq2bvqf2). We repeated analyses including only individuals with a baseline CDR-SB score of 0.50 (n = 66), and observed that restricting the sample to individuals with early state MCI did not lead to less variation at 18-month follow-up (figure e2). We then studied whether sample size influenced the 95% range of expected effect sizes based on simulated datasets including n = 1,000, n = 2,000, n = 5,000, and n = 10,000 individuals. Overall, these results point out that by increasing sample sizes the 95% range effect sizes narrow systematically (figures e3–e5; corresponding clinical trial effects were included only when group size was comparable). Most observed group differences of recent clinical trials remained within the 95% ranges for all outcome measures when matched with simulated group sizes, except for the EMERGE findings for the CDR-SB and ADAS-Cog and EXPEDITION-3 findings for the CDR-SB and MMSE.

### AD Risk Factors Influencing Disease Progression

**Figure 4:**
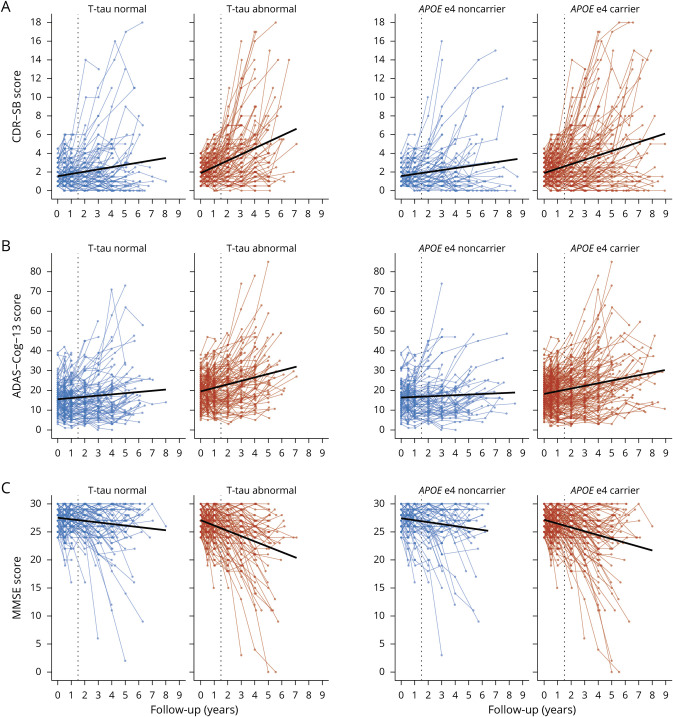
Individual Trajectories of Decline for Each Outcome Measure After Stratifying on Baseline Risk Factors Risk factors presented in the different rows for all outcome measures (A = Clinical Dementia Rating scale–sum of boxes [CDR-SB]; B = Alzheimer's Disease Assessment Scale–cognitive subscale [ADAS-Cog-13]; C = Mini-Mental State Examination [MMSE]).

**Figure 5:**
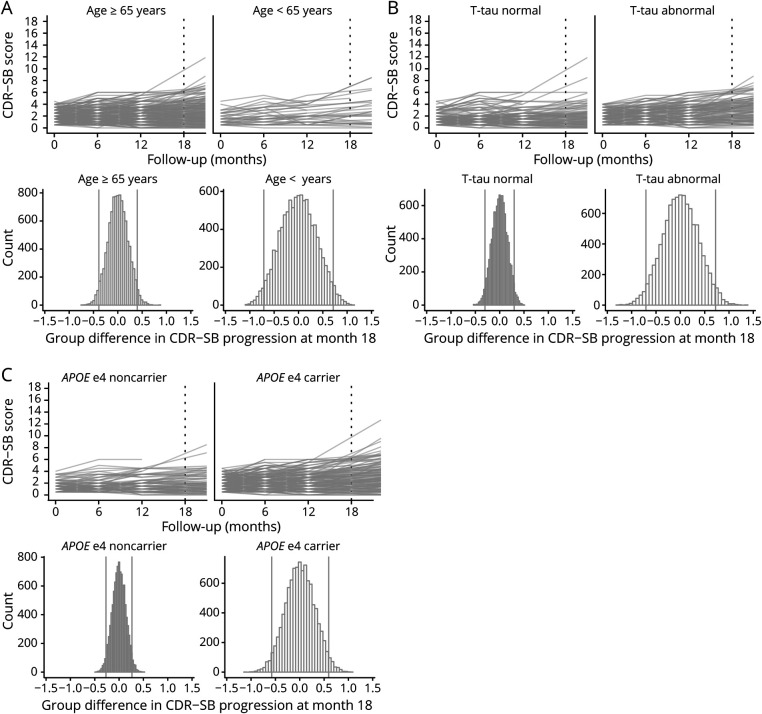
Individual Trajectories and Simulated 18-Month Group Differences for the Clinical Dementia Rating Scale–Sum of Boxes (CDR-SB) After Stratifying on Risk Factors

**Figure 6:**
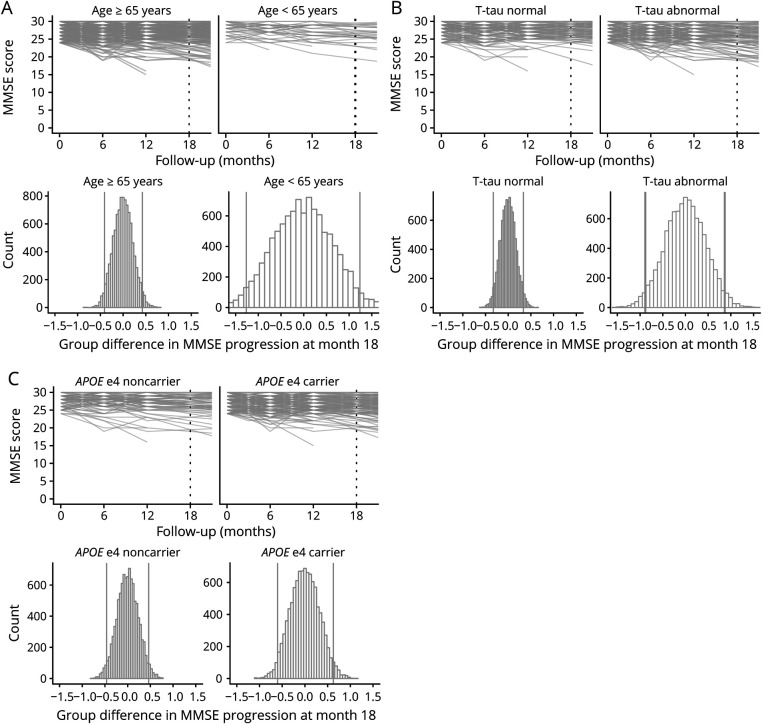
Individual Trajectories and Simulated 18-Month Group Differences for the Alzheimer's Disease Assessment Scale–Cognitive Subscale–13-Item Version (ADAS-Cog-13) After Stratifying on Risk Factors

**Figure 7:**
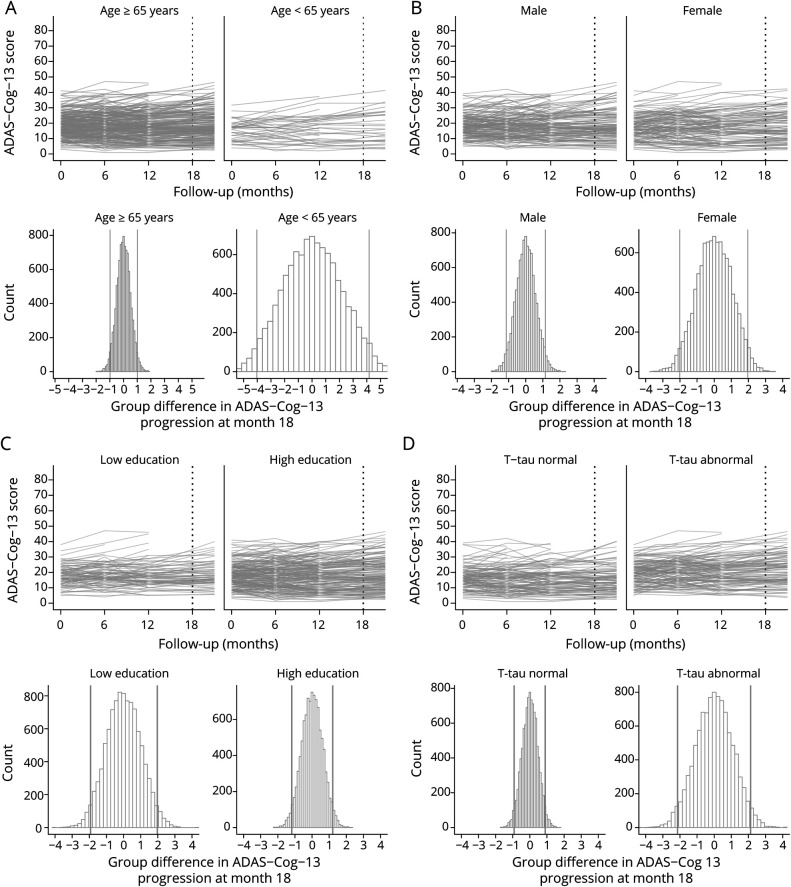
Individual Trajectories and Simulated 18-Month Group Differences for the Mini-Mental State Examination (MMSE) After Stratifying on Risk Factors

Figure 4 and e6 (doi.org/10.5061/dryad.qjq2bvqf2) shows the individual trajectories for each outcome measure by risk factor including all follow-up time points. Figures 5-7 and e7–e9 show the individual trajectories up to 18 months as well as histograms of simulated group differences at month 18 for each cognitive outcome measure, after splitting the overall group in high- and low-risk groups separately for each factor (i.e., baseline age, sex, educational level, *APOE* ε4 status, baseline CSF tau levels). In each figure, the left column represents the groups assumed to be at lower risk for fast progression, i.e., baseline age ≥65 (n = 261, 86%), male sex (n = 169, 56%), low education (n = 92, 30%), *APOE* ε4 noncarrier (n = 95, 31%), and normal tau levels (n = 123, 48%), whereas the right column represents the groups assumed to be at higher risk for fast disease progression (i.e., baseline age <65 [n = 41, 14%], female sex [n = 133, 44%], high education [n = 210, 70%], *APOE* ε4 carrier [n = 207, 69%], and abnormal tau levels [n = 135, 52%]).

For tau and *APOE*, overall decline over time was significantly steeper in the high-risk groups as compared to their low-risk counterparts, for all outcome measures (all time × risk factor interaction terms *p* values < 0.001) (figure 4). For age, sex, and educational level, decline over time did not differ between the high- and low-risk groups (figure e6, doi.org/10.5061/dryad.qjq2bvqf2).

Figure e7 (doi.org/10.5061/dryad.qjq2bvqf2) shows that for sex and educational level, the overall pattern of decline over 18 months was similar in both the high-risk groups and low-risk groups (i.e., being male vs being female, having lower vs higher education) and the 95% ranges of CDR-SB progression for the high-risk groups were largely similar to their low-risk counterparts. However, age, tau, and *APOE* high-risk groups (i.e., a baseline age <65 years, *APOE* ε4 carriers, baseline abnormal tau levels) showed a somewhat steeper decline over 18 months as compared to their lower risk counterparts (figure 5). Also, the 95% ranges of group differences were broader in those high-risk groups, showing that these risk factors lead to greater variability between individuals in CDR-SB progression. Similar results for age, tau, and *APOE* ε4 status were observed for the MMSE (figure 6 and figure e8). For the ADAS-Cog, a steeper decline over 18 months as well as greater variability between individuals was observed for the younger age <65, female sex, low education, and abnormal baseline tau groups (figure 7 and figure e9).

## Discussion

We investigated the influence of heterogeneity in disease progression among individuals with prodromal AD on effect sizes of commonly used cognitive outcome measures for AD trials. We found that individual trajectories on the CDR-SB, ADAS-Cog, and MMSE varied highly among individuals with prodromal and mild AD, even though the goal of inclusion criteria and subsequent group randomization is to create homogeneous groups. As a consequence, the 95% range of observed group differences on cognitive outcome measures at 18 months follow-up were broad, e.g., ranging from 0.35 points improvement to 0.35 points decline for the CDR-SB. Moreover, we showed that almost all group differences reported for recent anti-amyloid trials fell within this simulated 95% range of effect sizes for all outcome measures, i.e., meaning that they fell within the range of effect sizes that can be expected when there is actually no treatment effect. This suggests that, even though within some trials differences between placebo and treatment groups were statistically significant, the possibility cannot be excluded that those differences were actually due to oversampling of fast decliners in the placebo group or oversampling of slow decliners in the treated group. We further showed that, when repeating our simulation for separate risk factors associated with disease progression, a positive *APOE* ε4 status and baseline abnormal total tau levels were associated with steeper cognitive decline at a group level, but also with greater variability in progression. This resulted in even broader ranges of effect sizes in these high-risk groups on all outcome measures (e.g., ±0.70 points for the CDR-SB in those with baseline abnormal tau).

Our simulation reflects a clinical trial in which there is no real treatment effect, and so the 95% range of group differences between our “placebo” and “treatment” groups reflect the possible range of scores that can be expected when there is no treatment effect. Thus, this range of group differences can only be explained by heterogeneity between individuals in their cognitive decline. Comparing our simulation results with reported results from actual clinical trials revealed that these reported differences consistently fell within the 95% ranges of our simulation. So, although most trials showed an improvement of the treatment group vs the placebo group as captured by negative group differences, it is not possible to dissociate those results from chance findings due to unbalanced placebo vs treatment groups in terms of fast vs relatively slow decliners. This might even hold for the EMERGE trial, in which significantly less decline on the CDR-SB, ADAS-Cog, and MMSE were observed in the treated arm.^15^ Because those effects were not found in the identical ENGAGE trial, the possibility cannot be excluded that the “treatment effects” as observed in EMERGE could be due to random overrepresentation of slow decliners in the treatment group.

In most prodromal AD trials, amyloid-positive individuals are matched on *APOE* ε4 status, age, sex, and level of education, which are known to be associated with rate of disease progression.^4,5,18–20,27^ We also found that abnormal tau levels are associated with steeper decline at group level, as also suggested by previous studies.^4,19^ One strategy to overcome the problem of heterogeneity might thus be to further select on risk factors that are associated with rate of decline. However, when we tested this strategy, we indeed observed steeper decline in high-risk individuals at group level, but also more variability at subject level, as compared to low-risk individuals. Thus, “low-risk” individuals are likely to show a similar rate of relatively show progression, whereas, paradoxically, “high-risk” individuals still vary highly in their rate of progression. This implies that even a broader range of non-therapy-related effects are possible for the “high”-risk group, whereas in low-risk groups less variability among individuals is probably explained by the fact that all individuals show less decline. Consequently, only enrolling individuals at high risk of fast progression may not solve the issue of heterogeneity, but could increase heterogeneity, making it more difficult to capture a clinical effect on group level. Our findings illustrate that although at a group level individuals with both abnormal amyloid and tau show steeper decline, it is difficult to translate this to individual persons. This urges the further investigation of factors that can aid in prognostic estimates at an individual level.

Our results could be used to provide guidance on where to find “real” treatment effects, when heterogeneity in disease progression is unaccounted for. For example, the boundaries of the 95% range of effect sizes we found for the CDR-SB indicate that less decline of >0.5 points over 18 months would be unlikely due to random overrepresentation of fast decliners in this group. In other words, a decline of >0.5 points on the CDR-SB in the treated arm would strongly point towards a true clinical effect. A replication of this effect in a second trial would then be the ultimate proof of this clinical effect. Furthermore, the results from our simulation with samples ranging from n = 1,000 to n = 10,000 individuals highlight the influence of sample size on effects of heterogeneity, in that larger samples become less prone to imbalanced groups in terms of rate of decline. At the same time, this analysis shows that trials should be cautious with subgroup analyses, which diminishes group sizes and thereby increases the chance of finding a false-positive effect.

Other strategies for trials to deal with disease progression heterogeneity could be to investigate treatment effects on biomarkers for the intended target and expected downstream effects would further provide more information on treatment effects. This has for example has been done in the Dominantly Inherited Alzheimer's Network Trial Unit and the related gantenerumab trial, which demonstrated a treatment effect on markers of total tau, p-tau181, and neurofilament light.^28^ An advantage of biomarker outcomes compared to cognitive outcomes is that the former are less prone to day-to-day variability due to contextual factors (mood, concentration) or differences in practice effects due to repeated cognitive testing, and as such may show less heterogeneity in change over time across individuals. For a more direct demonstration of treatment effects on cognitive outcomes, it would be informative if trials examined individual cognitive trajectories rather than change on group level, to demonstrate whether individual tracts within the treatment group show less variability in decline than in the placebo group, which may point consistently to a treatment effect.

An additional strategy would be to incorporate a run-in test design, which could help to distinguish individuals who decline slowly from those who decline relatively fast. In the so-called “run-in period” all individuals would be “off-treatment,” and the cognitive change in this period might be a good estimate on the subsequent rate of disease progression.^29^ Still, a drawback of this approach is that long run-in periods are needed to capture cognitive changes, as our data as well as previous work suggest that currently used cognitive outcome measures do not adequately capture short-term changes in prodromal AD.^30^ Long run-in periods are undesirable, as they may increase the chance of attrition before the actual start of the trial. Future research should investigate whether alternative cognitive tests with improved sensitivity to detect subtle changes may shorten run-in periods. Several endeavors have been undertaken to develop more sensitive cognitive outcome measures for prodromal stages of AD, such as the Alzheimer's Disease Composite Score^31^ and the Cognitive–Functional Composite.^32^ Moreover, innovative cognitive tests that make use of digital technology and advanced scoring and assessment techniques^33^ are being developed, which may provide useful tools to capture more attenuation of disease progression more sensitively as well as reliably.^34,35^

There are some limitations that should be taken into account when interpreting our findings. First, our simulation was based on the ADNI database, a relatively highly educated community-based sample, with little diversity in terms of racial and ethnic characteristics. This has limited our investigation of heterogeneity in specific subgroups, particularly the young-onset group, and restricts the generalizability of our effect size boundaries to the global population. However, a strength of using the ADNI study is that it was designed to reflect a potential clinical trial population, hence the comparisons with recent clinical trial findings seem valid. A potential limitation regarding longitudinal cohort studies, such as ADNI, is attrition. To investigate potential attrition bias, we compared baseline characteristics of our selected cohort with a sample including all individuals who fulfilled all of our inclusion criteria without requirement of follow-up (as “intent-to-treat” cohort), and we found no differences between the two. Hence, it is unlikely that the intent-to-treat cohort would have shown a different rate in overall decline than our observed cohort. Another issue is that practice effects can arise when cognitive assessment are repeated over time, particularly at the second time of testing and when the time interval between assessments is short.^36^ In our ADNI selection, some participants had 6 months follow-up data, which may have led to less decline and thus less variability in change over time. In addition, it should be acknowledged that in general it is difficult to determine disease duration for individuals with AD at their first visit. Such variability in disease duration prior to study participation may have influenced variance in change at 18 months. Our approach to ensure a similar disease stage was by selection on baseline levels of cognitive functioning, as reflected by our inclusion criteria of an MMSE score of 24–30 and a global CDR score of 0.5. This is the only approach we have at the moment and is also used in clinical trials. So whereas it could be argued that an MMSE range from 24 to 30 is still somewhat broad, it should be noticed that this range has been common practice in recent and current clinical trials of prodromal AD.

Regarding our comparisons with recent clinical trials, it should be noted that our selection criteria and follow-up timeframe were based on the EMERGE and ENGAGE trials inclusion criteria,^17^ and that there may be small differences with the criteria used in the other trials with which we compared our findings. Furthermore, most clinical trials with which we compared our findings included up to 1,500 participants, whereas our original sample only included 300 participants. To enable a fairer comparison, we also ran our analyses with simulated datasets up to 5,000 participants. Finally, it should be taken into account that we investigated the influence of risk factors of disease progression by only looking at single risk factors, whereas accumulating evidence suggests that no one factor can explain heterogeneity in progression. Future research in large, independent datasets with extensive follow-up data like that in the ADNI study should be done to systematically test and validate an optimal prediction model for rate of disease progression, for example using machine learning tools.

Our study highlights the importance of understanding heterogeneity in AD progression in the context of clinical trials, by providing more insight on how this heterogeneity, if unaccounted for, could potentially affect trial outcomes. Our findings imply that only selecting individuals with abnormal amyloid and tau markers does not seem to solve the issue of heterogeneity, and in fact, may make it more difficult to capture potential treatment effects on clinical outcomes. Our findings may have use in determining thresholds for detecting (actual) treatment effects in prodromal AD and may thereby advance the successful evaluation of future clinical trials.

## Glossary

AD: Alzheimer disease
ADAS-Cog: Alzheimer's Disease Assessment Scale–cognitive subscale
ADAS-Cog-13: Alzheimer's Disease Assessment Scale–cognitive subscale–13-item version
ADNI: Alzheimer's Disease Neuroimaging Initiative
CDR: Clinical Dementia Rating
CDR-SB: Clinical Dementia Rating scale–sum of boxes
LMM: linear mixed model
MCI: mild cognitive impairment
MMSE: Mini-Mental State Examination
PiB: Pittsburgh compound B

## References

[1] Cummings J, Feldman HH, Scheltens P. The “rights” of precision drug development for Alzheimer's disease. Alzheimers Res Ther 2019;11:76. 31470905 10.1186/s13195-019-0529-5 PMC6717388

[2] Food and Drug Administration. Early Alzheimer's Disease: Developing Drugs for Treatment: Guidance for Industry. Food and Drug Administration; 2018.

[3] Fogel DB. Factors associated with clinical trials that fail and opportunities for improving the likelihood of success: a review. Contemp Clin Trials Commun. 2018;11:156-164. 30112460 10.1016/j.conctc.2018.08.001 PMC6092479

[4] van Rossum IA, Vos SJ, Burns L, et al. Injury markers predict time to dementia in subjects with MCI and amyloid pathology. Neurology. 2012;79(17):1809-1816. 23019259 10.1212/WNL.0b013e3182704056 PMC3475623

[5] Vos SJ, Verhey F, Frölich L, et al. Prevalence and prognosis of Alzheimer's disease at the mild cognitive impairment stage. Brain 2015;138(pt 5):1327-1338. 25693589 10.1093/brain/awv029 PMC5013930

[6] Jack CR Jr, Wiste HJ, Vemuri P, et al. Brain beta-amyloid measures and magnetic resonance imaging atrophy both predict time-to-progression from mild cognitive impairment to Alzheimer's disease. Brain. 2010;133(11):3336-3348. 20935035 10.1093/brain/awq277 PMC2965425

[7] Scheltens NME, Tijms BM, Heymans MW, et al. Prominent non-memory deficits in Alzheimer's disease are associated with faster disease progression. J Alzheimers Dis. 2018;65(3):1029-1039. 30103316 10.3233/JAD-171088 PMC6588161

[8] Deaton A, Cartwright N. Understanding and misunderstanding randomized controlled trials. Soc Sci Med. 2018;210:2-21. 29331519 10.1016/j.socscimed.2017.12.005 PMC6019115

[9] Doody RS, Raman R, Farlow M, et al.; Alzheimer's Disease Cooperative Study Steering Committee; Semagacestat Study Group. A phase 3 trial of semagacestat for treatment of Alzheimer's disease. N Engl J Med. 2013 Jul 25;369(4):341-50. doi: 10.1056/NEJMoa1210951. PMID: 23883379. 23883379

[10] Honig LS, Vellas B, Woodward M, et al. Trial of solanezumab for mild dementia due to Alzheimer's disease. N Engl J Med. 2018;378(4):321-330. 29365294 10.1056/NEJMoa1705971

[11] Salloway S, Sperling R, Fox NC, et al. Two phase 3 trials of bapineuzumab in mild-to-moderate Alzheimer's disease. N Engl J Med. 2014;370(4):322-333. 24450891 10.1056/NEJMoa1304839 PMC4159618

[12] Wessels AM, Tariot PN, Zimmer JA, et al. Efficacy and safety of Lanabecestat for treatment of early and mild Alzheimer disease: the AMARANTH and DAYBREAK-ALZ randomized clinical trials. JAMA Neurol. 2020;77(2):199-209. 31764959 10.1001/jamaneurol.2019.3988 PMC6902191

[13] Egan MF, Kost J, Voss T, et al. Randomized trial of Verubecestat for prodromal Alzheimer's disease. N Engl J Med. 2019;380(15):1408-1420. 30970186 10.1056/NEJMoa1812840 PMC6776078

[14] Vandenberghe R, Rinne JO, Boada M, et al. Bapineuzumab for mild to moderate Alzheimer's disease in two global, randomized, phase 3 trials. Alzheimers Res Ther. 2016;8(1):18. 27176461 10.1186/s13195-016-0189-7 PMC4866415

[15] Biogen. Biogen Q3 2019 Earnings Presentation. 2019. Accessed October 22, 2019. https://investors.biogen.com/static-files/5a31a1e3-4fbb-4165-921a-f0ccb1d64b65

[16] Siemers ER. Phase 3 solanezumab trials: secondary outcomes in mild Alzheimer's disease patients. Alzheimers Dement. 2016;12(2):110-120. 26238576 10.1016/j.jalz.2015.06.1893

[17] von Hehn C, von Rosenstiel P, Tian Y, et al. Baseline Characteristics from ENGAGE and EMERGE: Two Phase 3 Studies to Evaluate Aducanumab in Patients With Early Alzheimer's Disease (P4. 1-001). AAN Enterprises; 2019.

[18] Ferretti MT, Iulita MF, Cavedo E, et al. Sex differences in Alzheimer disease: the gateway to precision medicine. Nat Rev Neurol. 2018;14(8):457-469. 29985474 10.1038/s41582-018-0032-9

[19] Hersi M, Irvine B, Gupta P, Gomes J, Birkett N, Krewski D. Risk factors associated with the onset and progression of Alzheimer's disease: a systematic review of the evidence. Neurotoxicology. 2017;61:143-187. 28363508 10.1016/j.neuro.2017.03.006

[20] Martins CAR, Oulhaj A, de Jager CA, Williams JH. APOE alleles predict the rate of cognitive decline in Alzheimer disease. Neurology. 2005;65(12):1888-1893. 16380608 10.1212/01.wnl.0000188871.74093.12

[21] Rosen WG, Mohs RC, Davis KL. A new rating scale for Alzheimer's disease. Am J Psychiatry. 1984;141(11):1356-1364. 6496779 10.1176/ajp.141.11.1356

[22] Folstein MF, Folstein SE, McHugh PR. “Mini-mental state”: a practical method for grading the cognitive state of patients for the clinician. J Psychiatr Res. 1975;12(3):189-198. 1202204 10.1016/0022-3956(75)90026-6

[23] Williams MM, Storandt M, Roe CM, Morris JC. Progression of Alzheimer's disease as measured by clinical dementia rating sum of boxes scores. Alzheimers Dement. 2013;9(1 suppl):S39-S44. 22858530 10.1016/j.jalz.2012.01.005 PMC3660405

[24] Hughes CP, Berg L, Danziger WL, Coben LA, Martin RL. A new clinical scale for the staging of dementia. Br J Psychiatry. 1982;140:566-572. 7104545 10.1192/bjp.140.6.566

[25] Landau SM, Breault C, Joshi AD, et al. Amyloid-beta imaging with Pittsburgh compound B and florbetapir: comparing radiotracers and quantification methods. J Nucl Med. 2013;54(1):70-77. 23166389 10.2967/jnumed.112.109009 PMC3747730

[26] Shaw LM, Vanderstichele H, Knapik-Czajka M, et al. Cerebrospinal fluid biomarker signature in Alzheimer's disease neuroimaging initiative subjects. Ann Neurol. 2009;65(4):403-413. 19296504 10.1002/ana.21610 PMC2696350

[27] Kim YJ, Cho S-K, Kim HJ, et al. Data-driven prognostic features of cognitive trajectories in patients with amnestic mild cognitive impairments. Alzheimers Res Ther. 2019;11(1):10. 30670089 10.1186/s13195-018-0462-z PMC6343354

[28] ALZFORUM. In DIAN-TU, Gantenerumab Brings Down Tau. By a Lot. Open Extension Planned. Presented at: AAT-AD/PD 2020 Conference: Advances in Alzheimer's and Parkinson's Therapies. Accessed April 10, 2020. alzforum.org/news/conference-coverage/dian-tu-gantenerumab-brings-down-tau-lot-open-extension-planned.

[29] Frost C, Kenward MG, Fox NC. Optimizing the design of clinical trials where the outcome is a rate: can estimating a baseline rate in a run-in period increase efficiency? Stat Med. 2008;27(19):3717-3731. 18484598 10.1002/sim.3280

[30] Karin A, Hannesdottir K, Jaeger J, et al. Psychometric evaluation of ADAS-Cog and NTB for measuring drug response. Acta Neurol Scand. 2014;129(2):114-122. 23763450 10.1111/ane.12153

[31] Wang J, Logovinsky V, Hendrix SB, et al. ADCOMS: a composite clinical outcome for prodromal Alzheimer's disease trials. J Neurol Neurosurg Psychiatry. 2016;87(9):993-999. 27010616 10.1136/jnnp-2015-312383 PMC5013117

[32] Jutten RJ, Harrison JE, Brunner AJ, et al. The Cognitive-Functional Composite is sensitive to clinical progression in early dementia: longitudinal findings from the Catch-Cog study cohort. Alzheimers Dement. 2020;6(1):e12020. 10.1002/trc2.12020 PMC7164406 32313832

[33] Koo BM, Vizer LM. Mobile technology for cognitive assessment of older adults: a scoping review. Innov Agingr 2019;3(1):igy038. 10.1093/geroni/igy038 PMC6312550 30619948

[34] Sliwinski MJ, Mogle JA, Hyun J, Munoz E, Smyth JM, Lipton RB. Reliability and validity of ambulatory cognitive assessments. Assessment. 2018;25(1):14-30. 27084835 10.1177/1073191116643164 PMC5690878

[35] Bilder RM, Reise SP. Neuropsychological tests of the future: how do we get there from here? Clin Neuropsychol. 2019;33(2):220-245. 30422045 10.1080/13854046.2018.1521993 PMC6422683

[36] Jutten RJ, Grandoit E, Foldi NS, et al. Lower practice effects as a marker of cognitive performance and dementia risk: a literature review. Alzheimers Dement. 2020;12(1):e12055. 10.1002/dad2.12055 PMC7346865 32671181

